# Supplementary material for: Gall-ID: tools for genotyping gall-causing phytopathogenic bacteria
Source: PeerJ. 2016 Jul 19;4:e2222. doi: 10.7717/peerj.2222 (PMC4958008; doi:10.7717/peerj.2222)
Supplement: Table S1 [file peerj-04-2222-s001.pdf]

Supplementary Table 1. Comparison of Velvet to SPAdes assemblies of 14 isolates associated with crown gall.

	Velvet Assembly			SPAdes Assembly		
**Isolate name**	**# of scaffolds > 1kb**	**N50 (bp)**	**Assembly length (bp)**	**# of scaffolds > 1kb**	**N50 (bp)**	**Assembly length (bp)**
13-2099-1-2	52	275,131	5,552,211	36	305,993	5,655,819
13-626	579	21,870	6,842,659	47	520,764	6,958,258
AC27/96	154	96,285	7,277,651	62	304,507	7,294,256
AC44/96	47	329,597	6,092,333	42	411,299	6,103,740
B131/95	113	172,630	7,277,855	56	477,595	7,297,525
B133/95	78	301,459	7,163,747	57	478,752	7,169,549
B140/95	153	65,958	5,712,082	39	261,962	5,717,522
N2/73	52	299,053	5,744,801	28	583,418	5,768,133
W2/73	53	247,839	5,490,237	25	437,031	5,510,033
15-1187-1-2a	87	146,955	5,982,904	30	349,571	6,003,102
15-1187-1-2b	141	89,835	5,979,368	33	349,600	6,003,936
14-2641	168	101,816	6,253,349	NA	NA	NA
15-172	106	108,118	5,420,030	48	324,414	5,448,190
15-174	59	247,284	6,032,397	30	609,451	6,054,965
