# Supplementary material for: Gall-ID: tools for genotyping gall-causing phytopathogenic bacteria
Source: PeerJ. 2016 Jul 19;4:e2222. doi: 10.7717/peerj.2222 (PMC4958008; doi:10.7717/peerj.2222)
Supplement: Figure S1 — The 16S rDNA sequence from isolate originally labeled as Agrobacterium isolate 14-2641 was analyzed using the Phytopath-type tool. The isolate is labeled in red, as “query_isolate”; inset shows the clade that circumscribes the isolate. [file peerj-04-2222-s002.pdf]

SUPPLEMENTAL FIGURE 1

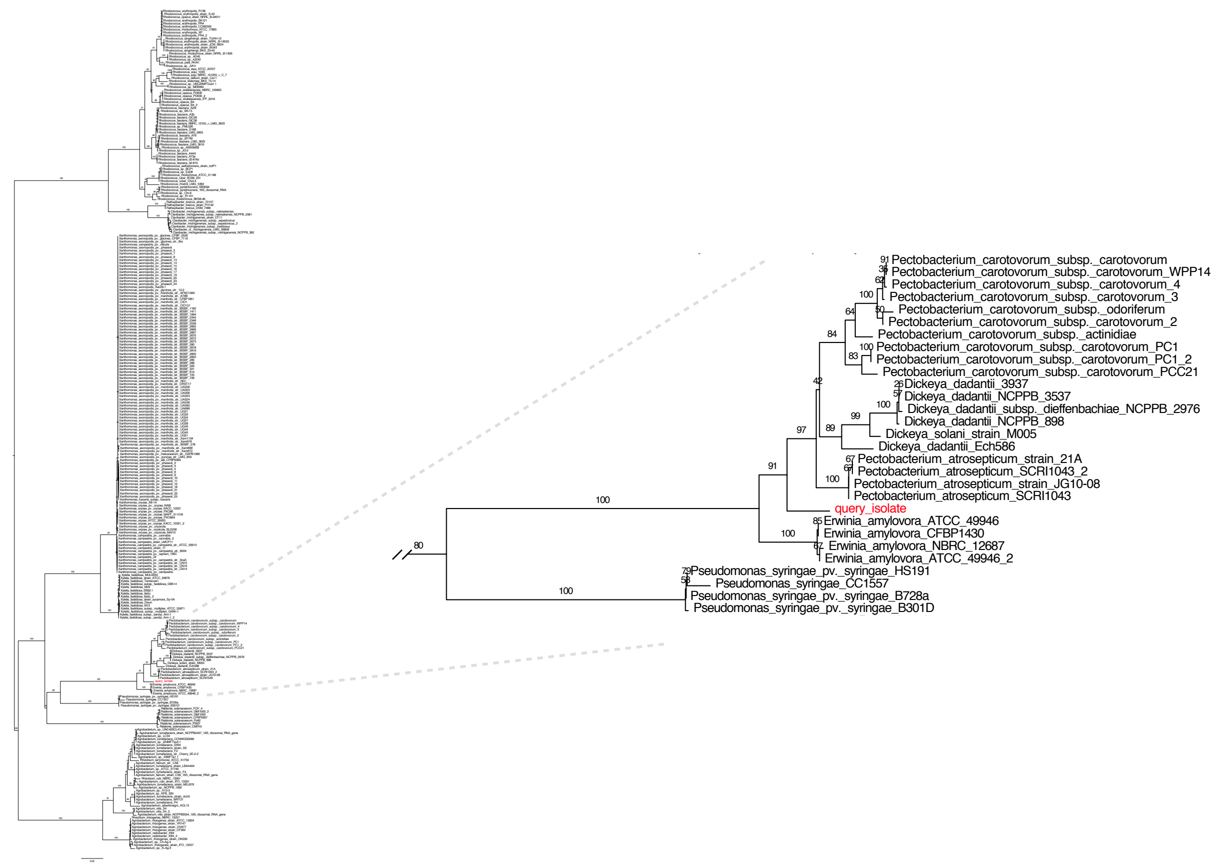

Supplemental Figure 1. Validation of the Phytopath-type tool. The 16S rDNA sequence from isolate originally labeled as *Agrobacterium* isolate 14-2641 was analyzed using the Phytopath-type tool. The isolate is labeled in red, as "query_isolate"; inset shows the clade that circumscribes the isolate.
