# Supplementary material for: Gall-ID: tools for genotyping gall-causing phytopathogenic bacteria
Source: PeerJ. 2016 Jul 19;4:e2222. doi: 10.7717/peerj.2222 (PMC4958008; doi:10.7717/peerj.2222)
Supplement: Figure S2 — Genome sequences assembled using Velvet v. 1.2.10 (blue) and SPAdes v. 3.7.0 (orange) were compared based on scaffold N50 and number of scaffolds greater than 1 kb in size. [file peerj-04-2222-s003.pdf]

SUPPLEMENTAL FIGURE 2

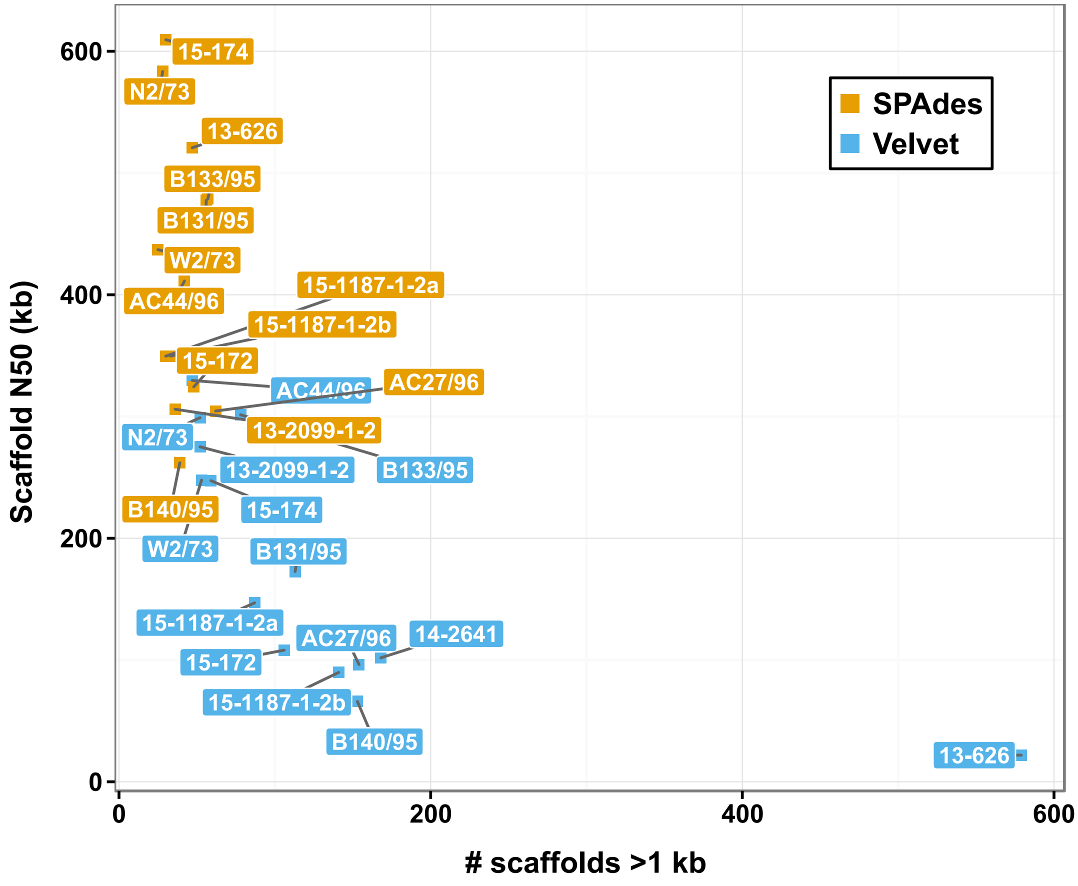

Supplemental Figure 2. The SPAdes assembler produces high quality assemblies. Genome sequences assembled using Velvet v. 1.2.10 (blue) and SPAdes v. 3.7.0 (orange) were compared based on scaffold N50 and number of scaffolds greater than 1kb in size.
