# Supplementary material for: Gall-ID: tools for genotyping gall-causing phytopathogenic bacteria
Source: PeerJ. 2016 Jul 19;4:e2222. doi: 10.7717/peerj.2222 (PMC4958008; doi:10.7717/peerj.2222)
Supplement: Figure S3 — The A. radiobacter K84 genome sequence (top) and assemblies of 13-626 generated using SPAdes v. 3.7.0 (middle; 47 scaffolds) and Velvet (bottom; 527 scaffolds) were aligned using Mauve. Shared locally collinear blocks (LCBs) between the reference and the two assemblies are color-coded and connected by color-coded lines. Scaffold and replicon ends are depicted as vertical red lines. [file peerj-04-2222-s004.pdf]

SUPPLEMENTAL FIGURE 3

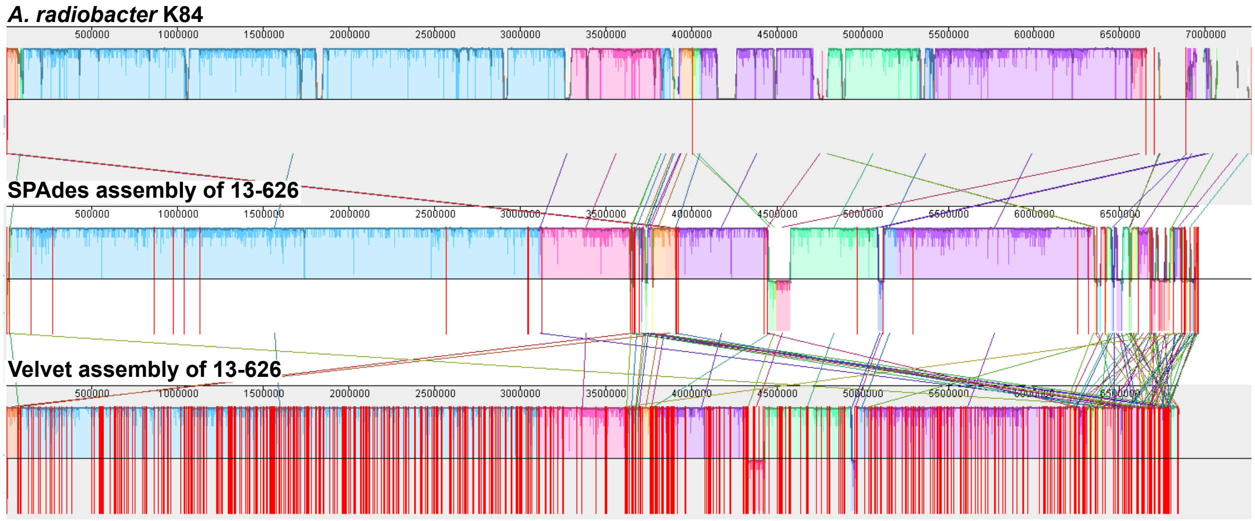

Supplemental Figure 3. Whole genome alignment of 13-626 assemblies to a close reference sequence indicates collinearity of genomes. The *A. radiobacter* K84 genome sequence (top) and assemblies of 13-626 generated using SPAdes v. 3.7.0 (middle; 47 scaffolds) and Velvet (bottom; 527 scaffolds) were aligned using Mauve. Shared locally collinear blocks (LCBs) between the reference and the two assemblies are color-coded and connected by color-coded lines. Scaffold and replicon ends are depicted as vertical red lines.
